# 3-[(2,4-Dichloro­phen­yl)imino­meth­yl]-2-hydr­oxy-5-methyl­benzaldehyde

**DOI:** 10.1107/S1600536809018303

**Published:** 2009-05-20

**Authors:** Işın Kılıç, Şamil Işık, Erbil Ağar, Ferda Erşahin

**Affiliations:** a Department of Physics, Faculty of Arts and Science, Ondokuz Mayıs University, TR-55139 Kurupelit-Samsun, Turkey; b Department of Chemistry, Faculty of Arts and Science, Ondokuz Mayıs University, 55139 Samsun, Turkey

## Abstract

The title compound, C_15_H_11_Cl_2_NO_2_, is a Schiff base which adopts the phenol–imine tautomeric form in the solid state, being stabilized by a strong intra­molecular O—H⋯N hydrogen bond. The mol­ecule is almost planar (r.m.s. deviation for all non-H atoms = 0.049 Å), displaying a dihedral angle of 3.1 (3)° between the planes of the two aromatic rings.

## Related literature

For Schiff bases as substrates in the preparation of number of biologically active compounds, see: Siddiqui *et al.* (2006 ▶). For photochromism and thermochromism in these compounds, see: Hadjoudis *et al.* (1987 ▶); Xu *et al.* (1994 ▶). For hydrogen-bond motifs, see: Bernstein *et al.* (1995 ▶). For related structures, see: Gül *et al.* (2007 ▶) Koşar *et al.* (2005 ▶).
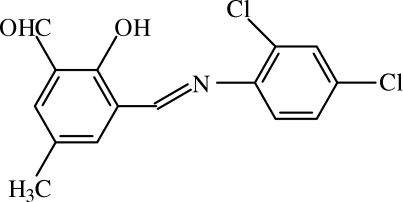


## Experimental

### 

#### Crystal data

C_15_H_11_Cl_2_NO_2_
*M* *_r_* = 308.15
Monoclinic, 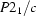

*a* = 19.424 (2) Å
*b* = 4.6113 (3) Å
*c* = 15.4245 (14) Å
β = 103.946 (8)°
*V* = 1340.9 (2) Å^3^
*Z* = 4
Mo *K*α radiation
μ = 0.48 mm^−1^
*T* = 296 K
0.80 × 0.31 × 0.02 mm

#### Data collection

Stoe IPDS II diffractometer
Absorption correction: integration (*X-RED*; Stoe & Cie, 2002 ▶) *T*_min_ = 0.817, *T*_max_ = 0.982
5912 measured reflections
2613 independent reflections
1228 reflections with *I* > 2σ(*I*)
*R*_int_ = 0.099

#### Refinement

*R*[*F* ^2^ > 2σ(*F* ^2^)] = 0.063
*wR*(*F* ^2^) = 0.138
*S* = 0.91
2613 reflections
183 parameters
H-atom parameters constrained
Δρ_max_ = 0.28 e Å^−3^
Δρ_min_ = −0.27 e Å^−3^

### 

Data collection: *X-AREA* (Stoe & Cie, 2002 ▶); cell refinement: *X-AREA*; data reduction: *X-RED32* (Stoe & Cie, 2002 ▶); program(s) used to solve structure: *SHELXS97* (Sheldrick, 2008 ▶); program(s) used to refine structure: *SHELXL97* (Sheldrick, 2008 ▶); molecular graphics: *ORTEP-3 for Windows* (Farrugia, 1997 ▶); software used to prepare material for publication: *WinGX* (Farrugia, 1999 ▶).

## Supplementary Material

Crystal structure: contains datablocks I, global. DOI: 10.1107/S1600536809018303/bt2960sup1.cif

Structure factors: contains datablocks I. DOI: 10.1107/S1600536809018303/bt2960Isup2.hkl

Additional supplementary materials:  crystallographic information; 3D view; checkCIF report

## Figures and Tables

**Table 1:** Hydrogen-bond geometry (Å, °)

*D*—H⋯*A*	*D*—H	H⋯*A*	*D*⋯*A*	*D*—H⋯*A*
O1—H1⋯N1	0.82	1.85	2.577 (4)	147

**Fig. 1.**
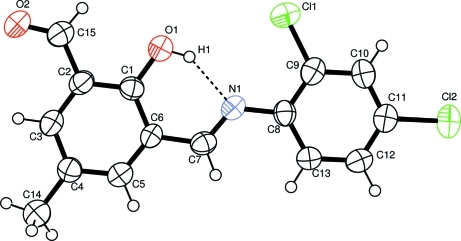
The molecular structure of (I), showing the atom-numbering scheme. Dashed lines indicate intramolecular hydrogen bond.

**Fig. 2.**
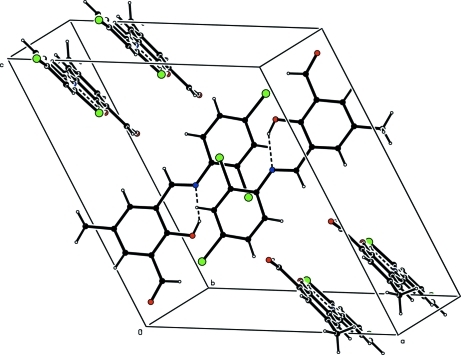
The crystal packing of compound (I). Dashed lines indicate intramolecular hydrogen bond.

**Fig. 3.**
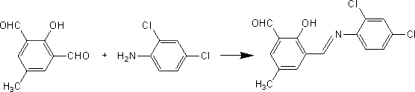
Synthetic scheme for C15H11Cl2O2N.

## supplementary crystallographic information

### Comment

In this paper, a new Schiff base, (I), C_15_H_11_Cl_2_O_2_N is prepared and its crystal structure is reported.

Schiff bases are synthesized from an aromatic amine and a carbonyl compound by a nucleophilic addition reaction. They are used as substrates in the preparation of number of biologically active compounds (Siddiqui *et al.*, 2006). Photochromism and thermochromism are also characteristics of these materials and arise *via* H-atom transfer from the hydroxy O atom to the N atom (Hadjoudis *et al.*, 1987; Xu *et al.*, 1994). These are two types of intra molecular hydrogen bonds in Schiff bases, in keto-amine (N—H···O) and phenol-imine (N···H—O) tautomeric forms. Our X-ray investigation shows that the title compound, (I), exists in the phenol-imine form.

An *ORTEP* view of the molecule of (I) is shown in Fig. 1. There is a strong O···N type intramolecular hydrogen bond and this intramolecular hydrogen bond (O1—H1···N1) can be described as an S(6) motif (Bernstein *et al.*, 1995). The C1—O1 [1.354 (6) Å] and C7=N1 [1.272 (6) Å] bond lengths verify the enol-imine form of (I), and these distances are agree with the corresponding distances in (*E*)-2-[4-(Dimethylamino)phenyliminomethyl]-6-methylphenol [1.350 (3) and 1.280 (2) Å; Gül *et al.*, 2007)] and in (*E*)-4-Methoxy-2- [(4-nitrophenyl)iminomethyl]phenol [1.351 (2) and 1.277 (2) Å; Koşar *et al.*, 2005]. The molecule is almost planar and the dihedral angle between the rings formed by atoms C1—C6 and C8—C13 is 3.12(0.28)°.

### Experimental

The compound 3-[(2,4-Dichlorophenyl)imino]methyl-2-hydroxy-5- methylbenzaldehyde was prepared by reflux a mixture of a solution containing 2-Hydroxy-5-methyl-1,3-benzenedicarboxaldehyde (0.05 g 0.3 mmol) in 20 ml e thanol and a solution containing 2,4-Dichloroaniline(0.049 g 0.3 mmol) in 20 ml e thanol. The reaction mixture was stirred for 1 hunder reflux. The crystals of 3-[(2,4-Dichlorophenyl)imino]methyl-2-hydroxy-5- methylbenzaldehyde suitable for X-ray analysis were obtained from ethylalcohol by slow evaporation (yield % 79; m.p.483–485 K).

### Refinement

All H atoms bound to carbon were refined using a riding model with C—H= 0.93Å and 0.96 Å *U*_iso_(H) = 1.2*U*_eq_(C_aromatic_) and C—H= 0.96Å *U*_iso_(H) = 1.5*U*_eq_(C_methyl_). The H atom of hydroxyl O atom was refined with O—H= 0.82Å *U*_iso_(H) = 1.5*U*_eq_(O).

### Crystal data

**Table:**

C_15_H_11_Cl_2_NO_2_	*F*(000) = 632
*M**_r_* = 308.15	*D*_x_ = 1.526 Mg m^−^^3^
Monoclinic, *P*2_1_/*c*	Mo *K*α radiation, λ = 0.71073 Å
Hall symbol: -P 2ybc	Cell parameters from 6202 reflections
*a* = 19.424 (2) Å	θ = 1.9–29.5°
*b* = 4.6113 (3) Å	µ = 0.48 mm^−^^1^
*c* = 15.4245 (14) Å	*T* = 296 K
β = 103.946 (8)°	Plate, red
*V* = 1340.9 (2) Å^3^	0.80 × 0.31 × 0.02 mm
*Z* = 4	

### Data collection

**Table:**

Stoe IPDS II diffractometer	2613 independent reflections
Radiation source: fine-focus sealed tube	1228 reflections with *I* > 2σ(*I*)
plane graphite	*R*_int_ = 0.099
Detector resolution: 6.67 pixels mm^-1^	θ_max_ = 26.0°, θ_min_ = 2.2°
rotation method scans	*h* = −23→22
Absorption correction: integration (*X-RED*; Stoe & Cie, 2002)	*k* = −5→5
*T*_min_ = 0.817, *T*_max_ = 0.982	*l* = −18→18
5912 measured reflections	

### Refinement

**Table:**

Refinement on *F*^2^	Primary atom site location: structure-invariant direct methods
Least-squares matrix: full	Secondary atom site location: difference Fourier map
*R*[*F*^2^ > 2σ(*F*^2^)] = 0.063	Hydrogen site location: inferred from neighbouring sites
*wR*(*F*^2^) = 0.138	H-atom parameters constrained
*S* = 0.91	*w* = 1/[σ^2^(*F*_o_^2^) + (0.0441*P*)^2^] where *P* = (*F*_o_^2^ + 2*F*_c_^2^)/3
2613 reflections	(Δ/σ)_max_ < 0.001
183 parameters	Δρ_max_ = 0.28 e Å^−^^3^
0 restraints	Δρ_min_ = −0.27 e Å^−^^3^

### Special details

**Table:**

Experimental. 120 frames, detector distance = 100 mm
Geometry. All e.s.d.'s (except the e.s.d. in the dihedral angle between two l.s. planes) are estimated using the full covariance matrix. The cell e.s.d.'s are taken into account individually in the estimation of e.s.d.'s in distances, angles and torsion angles; correlations between e.s.d.'s in cell parameters are only used when they are defined by crystal symmetry. An approximate (isotropic) treatment of cell e.s.d.'s is used for estimating e.s.d.'s involving l.s. planes.
Refinement. Refinement of *F*^2^ against ALL reflections. The weighted *R*-factor *wR* and goodness of fit *S* are based on *F*^2^, conventional *R*-factors *R* are based on *F*, with *F* set to zero for negative *F*^2^. The threshold expression of *F*^2^ > σ(*F*^2^) is used only for calculating *R*-factors(gt) *etc*. and is not relevant to the choice of reflections for refinement. *R*-factors based on *F*^2^ are statistically about twice as large as those based on *F*, and *R*- factors based on ALL data will be even larger.

### Fractional atomic coordinates and isotropic or equivalent isotropic displacement parameters (Å^2^)

**Table:**

	*x*	*y*	*z*	*U*_iso_*/*U*_eq_	
C1	0.1925 (3)	0.2637 (10)	0.3175 (3)	0.0447 (12)	
C2	0.1516 (3)	0.0564 (11)	0.2629 (3)	0.0489 (12)	
C3	0.0969 (3)	−0.0806 (10)	0.2900 (3)	0.0495 (13)	
H3	0.0702	−0.2192	0.2528	0.059*	
C4	0.0806 (2)	−0.0190 (10)	0.3702 (3)	0.0467 (12)	
C5	0.1227 (2)	0.1889 (10)	0.4239 (3)	0.0472 (12)	
H5	0.1134	0.2321	0.4789	0.057*	
C6	0.1770 (2)	0.3330 (9)	0.3998 (3)	0.0410 (11)	
C7	0.2183 (3)	0.5467 (10)	0.4580 (3)	0.0463 (12)	
H7	0.2062	0.5931	0.5110	0.056*	
C8	0.3140 (2)	0.8812 (9)	0.4948 (3)	0.0435 (11)	
C9	0.3702 (3)	1.0022 (10)	0.4659 (3)	0.0493 (13)	
C10	0.4154 (3)	1.2028 (10)	0.5178 (3)	0.0538 (14)	
H10	0.4531	1.2807	0.4980	0.065*	
C11	0.4039 (3)	1.2842 (10)	0.5983 (3)	0.0496 (13)	
C12	0.3487 (3)	1.1723 (10)	0.6292 (3)	0.0508 (13)	
H12	0.3419	1.2297	0.6843	0.061*	
C13	0.3035 (3)	0.9738 (10)	0.5774 (3)	0.0500 (13)	
H13	0.2656	0.9003	0.5976	0.060*	
C14	0.0213 (3)	−0.1709 (12)	0.3997 (3)	0.0618 (15)	
H14A	−0.0188	−0.0434	0.3918	0.093*	
H14B	0.0370	−0.2233	0.4615	0.093*	
H14C	0.0079	−0.3426	0.3644	0.093*	
C15	0.1671 (3)	−0.0215 (13)	0.1781 (3)	0.0676 (16)	
H15	0.2055	0.0712	0.1638	0.081*	
Cl1	0.38664 (8)	0.8931 (3)	0.36584 (9)	0.0671 (5)	
Cl2	0.46096 (7)	1.5343 (3)	0.66358 (9)	0.0634 (5)	
N1	0.2710 (2)	0.6740 (8)	0.4391 (2)	0.0464 (10)	
O1	0.24635 (18)	0.3927 (7)	0.2904 (2)	0.0581 (10)	
H1	0.2660	0.5101	0.3282	0.087*	
O2	0.1350 (2)	−0.1956 (9)	0.1242 (2)	0.0785 (12)	

### Atomic displacement parameters (Å^2^)

**Table:**

	*U*^11^	*U*^22^	*U*^33^	*U*^12^	*U*^13^	*U*^23^
C1	0.046 (3)	0.044 (3)	0.045 (3)	0.000 (2)	0.012 (2)	0.003 (2)
C2	0.051 (3)	0.056 (3)	0.039 (2)	0.002 (3)	0.010 (2)	−0.004 (2)
C3	0.055 (3)	0.050 (3)	0.041 (3)	−0.002 (2)	0.006 (2)	−0.005 (2)
C4	0.043 (3)	0.047 (3)	0.048 (3)	0.003 (2)	0.009 (2)	0.000 (2)
C5	0.050 (3)	0.049 (3)	0.042 (2)	0.005 (2)	0.010 (2)	−0.001 (2)
C6	0.047 (3)	0.037 (3)	0.037 (2)	0.001 (2)	0.006 (2)	−0.003 (2)
C7	0.055 (3)	0.044 (3)	0.041 (2)	0.005 (3)	0.015 (2)	0.004 (2)
C8	0.045 (3)	0.037 (3)	0.046 (3)	−0.001 (2)	0.005 (2)	0.002 (2)
C9	0.057 (3)	0.041 (3)	0.046 (3)	0.005 (3)	0.004 (2)	0.000 (2)
C10	0.052 (3)	0.045 (3)	0.061 (3)	−0.005 (2)	0.008 (3)	0.000 (2)
C11	0.052 (3)	0.043 (3)	0.049 (3)	0.003 (2)	0.002 (2)	0.001 (2)
C12	0.058 (3)	0.052 (3)	0.045 (3)	0.005 (3)	0.017 (2)	−0.001 (2)
C13	0.053 (3)	0.055 (3)	0.044 (3)	−0.006 (3)	0.017 (2)	−0.003 (2)
C14	0.063 (3)	0.062 (4)	0.062 (3)	−0.003 (3)	0.018 (3)	−0.007 (3)
C15	0.063 (3)	0.089 (4)	0.054 (3)	−0.012 (3)	0.021 (3)	−0.018 (3)
Cl1	0.0730 (9)	0.0789 (10)	0.0548 (7)	−0.0040 (8)	0.0263 (7)	−0.0082 (7)
Cl2	0.0617 (8)	0.0510 (8)	0.0691 (8)	−0.0052 (7)	−0.0007 (7)	−0.0080 (7)
N1	0.053 (2)	0.046 (2)	0.039 (2)	0.003 (2)	0.0093 (19)	−0.0035 (18)
O1	0.061 (2)	0.063 (2)	0.0516 (19)	−0.0117 (18)	0.0160 (17)	−0.0102 (17)
O2	0.086 (3)	0.101 (3)	0.052 (2)	−0.028 (2)	0.023 (2)	−0.026 (2)

### Geometric parameters (Å, °)

**Table:**

C1—O1	1.354 (6)	C8—N1	1.416 (5)
C1—C2	1.390 (6)	C9—C10	1.390 (6)
C1—C6	1.411 (7)	C9—Cl1	1.725 (5)
C2—C3	1.384 (7)	C10—C11	1.366 (7)
C2—C15	1.457 (7)	C10—H10	0.9300
C3—C4	1.378 (7)	C11—C12	1.374 (7)
C3—H3	0.9300	C11—Cl2	1.742 (5)
C4—C5	1.395 (6)	C12—C13	1.382 (6)
C4—C14	1.510 (7)	C12—H12	0.9300
C5—C6	1.371 (6)	C13—H13	0.9300
C5—H5	0.9300	C14—H14A	0.9600
C6—C7	1.440 (6)	C14—H14B	0.9600
C7—N1	1.272 (6)	C14—H14C	0.9600
C7—H7	0.9300	C15—O2	1.215 (5)
C8—C9	1.391 (7)	C15—H15	0.9300
C8—C13	1.404 (7)	O1—H1	0.8200
			
O1—C1—C2	119.2 (4)	C10—C9—Cl1	118.8 (4)
O1—C1—C6	121.8 (4)	C8—C9—Cl1	119.9 (4)
C2—C1—C6	119.1 (5)	C11—C10—C9	119.2 (5)
C3—C2—C1	120.0 (4)	C11—C10—H10	120.4
C3—C2—C15	120.0 (4)	C9—C10—H10	120.4
C1—C2—C15	120.0 (5)	C10—C11—C12	121.6 (5)
C4—C3—C2	122.4 (4)	C10—C11—Cl2	119.1 (4)
C4—C3—H3	118.8	C12—C11—Cl2	119.3 (4)
C2—C3—H3	118.8	C11—C12—C13	119.3 (5)
C3—C4—C5	116.5 (5)	C11—C12—H12	120.4
C3—C4—C14	122.1 (4)	C13—C12—H12	120.4
C5—C4—C14	121.4 (5)	C12—C13—C8	120.9 (5)
C6—C5—C4	123.4 (5)	C12—C13—H13	119.5
C6—C5—H5	118.3	C8—C13—H13	119.5
C4—C5—H5	118.3	C4—C14—H14A	109.5
C5—C6—C1	118.6 (4)	C4—C14—H14B	109.5
C5—C6—C7	120.8 (4)	H14A—C14—H14B	109.5
C1—C6—C7	120.5 (5)	C4—C14—H14C	109.5
N1—C7—C6	122.1 (5)	H14A—C14—H14C	109.5
N1—C7—H7	118.9	H14B—C14—H14C	109.5
C6—C7—H7	118.9	O2—C15—C2	126.4 (5)
C9—C8—C13	117.8 (4)	O2—C15—H15	116.8
C9—C8—N1	118.0 (4)	C2—C15—H15	116.8
C13—C8—N1	124.2 (5)	C7—N1—C8	124.2 (4)
C10—C9—C8	121.2 (5)	C1—O1—H1	109.5
			
O1—C1—C2—C3	179.1 (4)	C13—C8—C9—C10	1.5 (7)
C6—C1—C2—C3	−0.6 (7)	N1—C8—C9—C10	−178.8 (4)
O1—C1—C2—C15	0.5 (7)	C13—C8—C9—Cl1	179.0 (3)
C6—C1—C2—C15	−179.2 (4)	N1—C8—C9—Cl1	−1.3 (6)
C1—C2—C3—C4	0.2 (7)	C8—C9—C10—C11	−0.7 (7)
C15—C2—C3—C4	178.9 (4)	Cl1—C9—C10—C11	−178.2 (4)
C2—C3—C4—C5	−0.5 (7)	C9—C10—C11—C12	0.0 (7)
C2—C3—C4—C14	−179.5 (4)	C9—C10—C11—Cl2	179.7 (3)
C3—C4—C5—C6	1.2 (7)	C10—C11—C12—C13	−0.3 (7)
C14—C4—C5—C6	−179.8 (4)	Cl2—C11—C12—C13	−180.0 (4)
C4—C5—C6—C1	−1.5 (7)	C11—C12—C13—C8	1.2 (7)
C4—C5—C6—C7	179.7 (4)	C9—C8—C13—C12	−1.8 (7)
O1—C1—C6—C5	−178.5 (4)	N1—C8—C13—C12	178.5 (4)
C2—C1—C6—C5	1.2 (6)	C3—C2—C15—O2	2.4 (9)
O1—C1—C6—C7	0.3 (7)	C1—C2—C15—O2	−179.0 (5)
C2—C1—C6—C7	180.0 (4)	C6—C7—N1—C8	−178.7 (4)
C5—C6—C7—N1	176.8 (4)	C9—C8—N1—C7	179.7 (4)
C1—C6—C7—N1	−2.0 (7)	C13—C8—N1—C7	−0.6 (7)

### Hydrogen-bond geometry (Å, °)

**Table:**

*D*—H···*A*	*D*—H	H···*A*	*D*···*A*	*D*—H···*A*
O1—H1···N1	0.82	1.85	2.577 (4)	147
